# Address

**Published:** 1888-07

**Authors:** W. H. Atkinson


### THE DENTAL REGISTER.

Vol. XLII.] JULY, 1888. [No. 7.

### Communications.

### Address.

BY W. H. ATKINSON, M.D., D.D.S.

(Delivered before the graduating class of the Indiana Dental College, March 7th, 1888.)

To understand the responsibility of any professional calling necessary to the well-being of society, it is important to comprehend the possibilities for good involved in its study of principles and practice.

As the teeth are the last of the organs of the body to attain maturity, it is evident that their potency for good and for evil to the whole organism is paramount.

As the organs have to be formed before they can perform the duty with which they are charged, the plan and mode of production must be understood, to enable us to judge of their quality and character and apportion the measure of the onus of work.

Teeth being situated at the portal of the digestive apparatus and constituting its primal link, justifies their claim to careful attention, and closest scrutiny, and investigation, as to origin, longevity and completion of career properly belonging to them as organs of expression, mastication and speech. To comprehend the nature of any apparatus belonging to the human system of organs it is necessary to know their origin and growth to perfection of structure.

The status of any calling is commensurate with the knowledge possessed by those who are its representatives.

The appreciation of a calling depends upon the will of those who employ those who practice the specialty.

The will is guided by knowledge or prejudice, whichever prevails among the people. These sometimes teach the same doctrine but they may also be at direct or implied contradiction of statement in text and meaning.

When the range of knowledge is very limited, controversies are sharp, severe and positive, in the ratio of limitation of the doctrine and text. But where erudition is sufficiently replete to enable us to perceive that as yet we know but in part, disputation and polemic contention will be resolved into legitimate discussion in which each proposition will be honestly and fairly stated, urged and defended to acceptance or rejection by those engaged in the noble effort to discover, maintain and practice the truth as far as known, and thus await further revelation upon these questions that are "held under judgment" until satisfactorily settled to all interested in their solution and establishment as "sound doctrine."

What has dentistry done and what must it yet do for those who suffer from false teaching in physiology, pathology, therapy, and hygienic instructions?

First, it has abolished ambiguity of diagnosis; polypharmacy in prescription and opened the way to the better understanding of wholesome feeding which when properly formulated, taught and practised, will inaugurate soundness of pabulum from which only physiological action can arise; and hence no possibility of aberration in functional activity.

The work that remains to be done is to review and pursue to satisfactory demonstration the already well advanced histological and embryological research, surely leading to this much desired and all important demonstration of function and factor of function lying at the bottom of physiology and its minifications in pathology, demanding therapeutical management to restore the lost balance in functional activities, which loss of balance has figured as diseases in the manifold manisfestations of weakness, deflection and destruction of function.

When the inquiry is made "how came dentistry to be a necessity to the people," it opens a very unpleasant investigation respecting the manner in which the teachers of the past have conducted themselves in investigations of what constitutes the well-being of the race.

In looking over investigations into the anatomy of the human body it will be discovered that the completed organs were the subject of inquiry to the exclusion of the alternations of generation of tissue elements out of which all the organs of the body take their origin.

This neglect of the intricate study of the embryological relations of all the organs of the body and the provisional character of the foresteps, as precedent necessities of well organized structures, was ignored in the investigations as well as in the literature that constitute the text of medical doctrine. This neglect induced unhygienic habits, resulting in imperfectly developed teeth, which led to such suffering as ultimately to demand attention to these neglected parts of the human system.

For the last half century investigations have been made which are fast leading to a better understanding of how the teeth are formed and maintained in working order. If we were to ask "what the mission of dentistry is" and "how well it has succeeded in practicalization of that mission" we are justified in the claim that it has done more to rectify misapprehensions in anatomy, physiology, pathology and therapy than any of the precedent studies, being as it is the culmination and ripened fruit of all the precedent efforts at comprehending the significance of all organized bodies as a part of functioning machinery, the completeness of which perfects the apparatus through the exercise of which, complete digestion and assimilation are attained.

Dentistry in the highest expression prevents destruction of the masticatory apparatus.

To this it now can only be said to be looking *forward*, as the final result of the investigations involved in a complete understanding of its mission.

It has already attained great excellence in the restoration of lost portions of teeth by filling, and lost members of the apparatus by substitution of efficient representatives of the natural organs, and we even now may say that legitimate exercise of the function of the dentist has well nigh annihilated the miseries of middle and advanced age by keeping the masticatory apparatus in such condition as to properly prepare the food for digestion, perfection of which results in the formation of perfect peptones supplying the blood column with euplastic pabulum for the nourishment of all the tissues, thus warding off the discrasia, that is a precedent necessity to the pathological status. It is well known that individuals who are in possession of full health by reason of the full performance of all the functions of the body are proof against infectious and contagious forms of specific diseases, and those who claim to comprehend the subject have tacitly admitted the doctrine just named by the advice they give their students and practitioners to always take a lunch before visiting patients suffering from epidemics of the character named.

To which we would add with emphasis, if we wish to shut out the contagion, "see to it that the horny layer of the epithilium constituting the surface of the body be intact, and if any abrasions of any exposed portions of the skin can be discovered let it be protected by some impervious substance well disinfected, such as court-plaster, isinglass plaster, collodion, or whatever may be at hand to seal up the fractured or abraded place." The comprehension and justification of the reasons for this advice involves an understanding of the molecular affinities beyond that embodied in text-books extant.

The greatest hinderance in the way of presenting professional subjects to a mixed audience grows out of the technical manner resorted to in professional schools of unnecessarily obscuring the meaning to all but those specially instructed in technical terms. Any one who has clear apprehensions of the subject matter of his discourse may easily find language to communicate any matter he has reduced to the status of knowledge. Divinity, law and medicine are to this day overlaid in their text-books by the wretchedness of technicality unnecessarily employed and when we speak to the advocates of this method they assume a squeamish delicacy by saying it would be immodest to call attention to the various parts of the body by a name that all would understand

As children we were taught that we only had head, stomach and heels, and were thus necessarily limited in making known our sufferings to our nurses. Some of the old Quakers did allow their children to have belly-ache, but it was doubtful whether *that* should not have been stomach-ache in view of the anatomical nominations allowed.

By reason of this technicality and squeamishness all distinct knowledge of teeth other than that they were the source of much suffering found little mention in anatomical works in English until Goodsir of England took upon himself deeper investigations into the origin and significance of these organs. It is no wonder then that the practice of dentistry so long principally consisted in the extraction of teeth. It was common for graduates in medicine, previous to the last thirty years, to ignore the teeth as undeserving of other than extirpative treatment, and of course the families under their advisement have followed their lead until artificial substitutes in the younger States became almost a universal necessity. Within twenty-five years the wide spread destruction of teeth in Indiana, Illinois and other Western States was so great as to present numerous examples of mothers in their teens with entire dentures of artificial construction, sometimes upon the upper jaw alone, but frequently involving both jaws. The practice of parapetetic tooth extractors travelling from house to house and ruthlessly removing the teeth from all whom they could persuade to submit to their treatment was followed by others in a short time who inserted teeth that "would never ache" according to the statement of the learned dentist. This practice prevailed not only in by-places where there were no religious teachers or limbs of the law, but directly under the noses and in the families of the so-called scholarly professions and it is painful to add that the medical fraternity cannot be exculpated from a like indifference to the well-being of the mouths of the patients for whose general maladies they were continually prescribing. In fact it is more difficult to meet the views of patients assuming a knowledge of their teeth they do not possess than those who have simply learned that there is great advantage derived from employing the dentist. As a rule the majority of patients to this day think that if they can preserve their front teeth so as to be presentable in looks that that should limit their efforts at preservation.

It might be thought that this statement only held good in by-places,and thinly peopled territories. Observation teaches otherwise.

Where people have inherited good teeth themselves they are apt to neglect their children's teeth until too late to secure the best results. Where *they* have suffered by reason of frail teeth, as a rule they will take their children at an early age to the dentist. The mistake of teachers lies in the impliedly final manner of communicating what they think they know, as if the whole round of knowledge of the subject had been settled; which is true only of the relations of numbers which when once known forever remain as sure foundation for all calculations in mathematical and mechanical researches as precedent steps in efforts attempting solution of body and process; evolutions of which constitute anatomy and physiology in partial and complete manifestation of health, sickness and restoration or destruction of function and factor of function in cognizable presence. Types of body and function are like the English verb subject to regular, irregular and defective modes of presentment. The regular lead, conforming to ritualistic plan for action; the irregular to impact from outside current inducing other activity than that within ritualistic limitations; and the defective, to arresting, minifying or deflecting current preceding imperfection in body and function, thus inducing variation of body and novelty in function to the mystification of all who rely upon set rules for the revelation of correct doctrines in anatomy, physiology, pathology and therapy, all of which are in the advance to better apprehension and higher appreciation step by step as men learn to cast off effete previous methods of study and take on the surer mode of advancement to truth by the mutually interrogative way of direct questioning each of the other when striving for satisfactory statement of the truth as it is. In coarse or mass anatomy as represented in sculpture and the painters' art the Greeks have not yet been equalled, but in embroyological and histological discovery and representation we are much in advance of them.

This is specially referred to in this place for the purpose of accentuating the trend of the effort to vindicate the doctrine of *progress* in *science* from all its phases as presented in the old methods of claimed final attainment in divinity, law, medicine and the classics, made up of mathematics, arithmetic, rhetoric, language and grammar.

The progress made in these departments of learning in a general way may be said to have advanced in an inverse order as just stated, viz.: grammar, medicine, law and divinity.

It was natural for learners to discredit and attack statements which seemed to them inadequate, deficient or false in the least sacred department first.

And as they conquered the set rules and inaugurated better methods of expression in this little esteemed authorization and learned that the anathema of the teachers did not annihilate them, they began to investigate and criticise the other departments until at length the voice of the people became the voice of God. "Vox populi, vox Dei" of the Latins. Thus was the right of private judgment of the learner established, and individual growth no longer depended on the rule and the rod of the pedagogue for the explanation and establishment of truth in the mind of the pupil. Growth of mind like the growth of body, was found to depend upon that upon which it fed, and thus education became "drawing out" and not a driving in mode of instruction.

In and struo are the roots of our word instruction. There must be a foundation on which to build, when the materials are brought to the place where the structure is to be raised. The character of foundation and material, mark the limitations which measure the possibilities of the future edifice.

Consciousness *per se* is the source and ground of all forms of segregate individual conscious perception through which we become aware of our mental and bodily existence and style of being, whereby we discriminate between the I and the not I, or the I and the other in the effort to comprehend ourselves and the foundation on which we stand.

In, within, and struo, I build, signifies inbuilding of mind and of body upon the foundation of conscious substance, the infinite, eternal shining of whose radiance is infinitesimally caught by arrest, inflection and storing involvement producing infinitesimal entities of mind and body as first postulates of being having irradiant career; societary arrangements of which upon a definite plan constitute worlds and their inhabitants in classifiable order for study, by careful pursuit of which truth is revealed. In the pursuit of truth as of love it may be said as the poet has it: "All love the purchase, *few* the price will pay, *love* only is the price of love."

He who seeks truth for her own sake at all hazards will not be overborne of much company in his early attempts at her acquisition; nevertheless the riches of such treasure are so soul satisfying with the luminosity of joy and peace that the effort is commendable even at the cost of every other preferment. It is a good thing to be on the right road to knowledge, but it is a better thing to be sure you are with face to the front, the best testimony of which is the strenuous opposition of old fogy professors and leaders, who, if you hope to benefit, you will find, as the poet says:

"Men must be taught as if you taught them not.
And things unknown proposed as things forgot."

The best teacher is he who leads his pupils to discover the truth as they think without his textual statement of its exact proportions.

Study of traditional deductions recorded as history has led some to say, "as uncertain as statistics."

When endeavoring to utilize the observations of predecessors, tradition is liable to be forgotten, minified or perverted in statement by reason of feeble memory, inattention, moral obliquity or ignorance of those who feel bound to never reveal its tenets by sign, mark, letter or understandable symbol by which it may be known to those who do not receive it by word of mouth of him who is authorized to communicate it to novices, (priest, professor.)

To assume that there is a special medicine for each disease is to assume that special food only satisfies special hunger, which is true when we go deep enough to perceive that this resides in deficiency of tissue.

From the Esculapian claim of plenary divine endowment through Hypocratic lay empiricism, tradition, legend and charm, medicine has come by well observed and classified facts approaching scientific presentment of function and factor of function to utilization of the value in verity of each phase of its growth to a somewhat coherent basis of textual statement in physiology, pathology and therapy.

But medicine will not be a science until biological chemistry shall have settled the origin and manner of molecular changes through which protoplasm, proximate principles, micro-organisms, tissues, organs and systems of organs arise, capable of growth, maintenance and completion of function and career.

A learned lecturer recently said: "It is not every or any sort of knowledge that enables one to judge wisely in the selection of a medical attendant. One reason for this is the general ignorance of the history of the evolution of medicine into its best form of the present day, an evolution in the course of which nearly every possible mode of blundering and straying from the true path has been tried over and over again. It is not by theories but by long and patient observation and experience that we come to know of the practice of medicine, and it is only the man or woman who has by long study based on careful preliminary education mastered the results of all this work who is to be trusted as your physician." This is the "Nirvana" (blown out light) by which a brilliant course of lectures on the history of medicine is reported to have wound up!!

This is blatant egotism or a discouraging piece of information to be given to an expectant popular audience assembled in the hope of learning how to avoid the terrible calamity of making a fatal mistake in selecting the source of instruction to help them to maintain a sound and equable mind in a lithe well working body. By all means get the best advice you can.

Yes, but how shall we discriminate the best from the inferior and worst? Aye! "There's the rub." And this apostle of "preliminary education," "observation," "experience," "evolution" and "mastery" of "blundering" and attainment of "the results of all this work," leaves us in the slough of despond without specific "prescription" for our malady.

Here at least is a "blunder" for us to master. A blunder of omission on the part of the orator, or reporter of what was said.

When one in reach of the library containing the record of "all this work" of *mastering* of "blunder" of predecessors fails to give the talisman endowing his listeners with the power to discriminate in selecting their "medical attendant"—where are we to look for safety?

Evidently not to the record kept out of reach of heteropathic or homeopathic acceptance and appropriation, but to the best revelation we can get in our extremity by the vehement urge of our necessity, opening our minds to the mediate or immediate perception of the way out of difficulty which is sure to come to all who are in a truly asking or teachable state of mind and affection.

The mediate revelations come by memory, books or friends; the immediate from the radiant source of all information, impinging upon the stored radiance in our consciousness, we call the seat of perception, which, when satisfied controls the will and then we can act in agreement with both affection and clear intellection.

When patients ask how to select professional attendants tell them to rely upon the advice of those they can trust when such can be had. And when among strangers to talk with the professional on some subject with which you are familiar, and if they show good sense in that then you may trust them in their special line to give you a good service. In case the measure of intelligence is not satisfactory, then pay their fee and look further, thanking your stars for safe deliverance at least from present danger of being in the care of an incompetent professional adviser.

Most professionals are exposed to talking either not enough or dazing you with too much talk.

Cold, dignified reticence repels more patients than radiant gushing interest even to undue freedom taken in their sufferings. these are the mistakes of beginners or those who never should have taken the responsibilities of professional life.

Deliberation is imperative; undue hesitancy is fatal to taking the right steps in the right manner and time to attain the best result; over timidity and modesty are as much to be avoided as over confidence, over boldness and roughness of manner.

The blood space of worlds and the lymph space of light,
The trysting place is where cooing and wooing
Of plus and of minus fore-shadows the night;
In restful remembrance of loves best brewing.

Orient nectar of Cupids free effort,
Attaching affections to bright wisdoms car;
The logos on going becomes now the support,
Of mind in its body thus standing at par.

Urge of the impact that asketh receiveth—
And thus is the type of the product fulfilled;
Divinely accordant product believeth,
And thus is completed what Creator willed.

The word of the mater and pater combined,
Result in the infinite system of suns;
In cycles and circles of planets entwining,
Unravelling of which is the greatest of puns.

The planets appearing in blood space is world,
While that in the lymph space is mere satellite;
The impact of radiance irradient hurled,
These stored irradiants and placed them aright.

To receive the afflatus—ether as breath,
As whirling thro' orbit they poles thus aquire;
Destruction of which produces their death,
And thus in chaotic surprize they expire.

